# Expression Profile and Gene Age Jointly Shaped the Genome-Wide Distribution of Premature Termination Codons in a *Drosophila melanogaster* Population

**DOI:** 10.1093/molbev/msu299

**Published:** 2014-11-03

**Authors:** Haiwang Yang, Bin Z. He, Huijing Ma, Shun-Chern Tsaur, Chenyu Ma, Ying Wu, Chau-Ti Ting, Yong E. Zhang

**Affiliations:** ^1^State Key Laboratory of Integrated Management of Pest Insects and Rodents & Key Laboratory of the Zoological Systematics and Evolution, Institute of Zoology, Chinese Academy of Sciences, Beijing, China; ^2^FAS Center for Systems Biology & Howard Hughes Medical Institute, Harvard University; ^3^Department of Mathematics and Science, National Taiwan Normal University, New Taipei City, Taiwan, Republic of China; ^4^State Key Laboratory of Plant Genomics and National Center for Plant Gene Research, Institute of Genetics and Developmental Biology, Chinese Academy of Sciences, Beijing, China; ^5^Department of Life Science, Genome and Systems Biology Degree Program, Institute of Ecology and Evolutionary Biology, Institute of Zoology, and Research Center for Developmental Biology and Regeneration Medicine, National Taiwan University, Taipei, Taiwan, Republic of China

**Keywords:** gene loss, premature termination codon, midgut, young gene, gene duplication

## Abstract

Widespread premature termination codon mutations (PTCs) were recently observed in human and fly populations. We took advantage of the population resequencing data in the *Drosophila* Genetic Reference Panel to investigate how the expression profile and the evolutionary age of genes shaped the allele frequency distribution of PTCs. After generating a high-quality data set of PTCs, we clustered genes harboring PTCs into three categories: genes encoding low-frequency PTCs (≤1.5%), moderate-frequency PTCs (1.5–10%), and high-frequency PTCs (>10%). All three groups show narrow transcription compared with PTC-free genes, with the moderate- and high-PTC frequency groups showing a pronounced pattern. Moreover, nearly half (42%) of the PTC-encoding genes are not expressed in any tissue. Interestingly, the moderate-frequency PTC group is strongly enriched for genes expressed in midgut, whereas genes harboring high-frequency PTCs tend to have sex-specific expression. We further find that although young genes born in the last 60 My compose a mere 9% of the genome, they represent 16%, 30%, and 50% of the genes containing low-, moderate-, and high-frequency PTCs, respectively. Among DNA-based and RNA-based duplicated genes, the child copy is approximately twice as likely to contain PTCs as the parent copy, whereas young de novo genes are as likely to encode PTCs as DNA-based duplicated new genes. Based on these results, we conclude that expression profile and gene age jointly shaped the landscape of PTC-mediated gene loss. Therefore, we propose that new genes may need a long time to become stably maintained after the origination.

## Introduction

Loss-of-function (LoF) mutations are generally believed to be deleterious and have been discussed in the context of human medicine for decades (Chang and Kan 1979; Rosenfeld et al. 1992; Frischmeyer and Dietz 1999). However, recent genome-wide surveys in human and fly populations revealed an unexpected prevalence of LoF mutations with hundreds or thousands of genes harboring deletions and/or premature termination codon mutations (PTCs) (Hoehn et al. 2012; Lee and Reinhardt 2012; MacArthur et al. 2012). Further analyses revealed that these genes are narrowly transcribed and encode high numbers of paralogs (Lee and Reinhardt 2012; MacArthur et al. 2012). The first feature suggests that these LoF-encoding genes may have relatively low pleiotropy, whereas the second feature indicates a certain extent of compensation. These two features might explain why the loss of these genes is tolerable (MacArthur et al. 2007, 2012).

Although these pioneering works provide the first genome-wide glimpse of LoF mutations and partially explain why these mutations are more prevalent than previously expected, the following two aspects remain to be explored. First, the allele frequencies of LoF variants have not been explicitly considered in evaluating the potential impact of the variants on gene function. We might expect that singleton or rare mutations are more likely to represent de novo mutations or mutations with deleterious effects, whereas moderate- or high-frequency mutations may be more tolerable and even beneficial. For example, the PTC-mediated loss of *CASPASE12* in humans may confer protection from severe sepsis, and this key mutation is nearly fixed in human populations (Wang et al. 2006). Therefore, genes harboring LoF mutations of different frequencies may show different functional and evolutionary features.

Second, the evolutionary age dependence of the probability of encoding LoF variants is not fully understood. In other words, how do young genes and old genes differ in their probability of containing LoF variants? What about new genes that originated through different mechanisms, for example, DNA-based duplication, RNA-based duplication or retroposition, and *de novo* origination (Kaessmann 2010; Zhang et al. 2012)? Even shortly after their origins, species-specific or lineage-specific genes may play versatile functions and serve as drivers of phenotypic evolution (Chen et al. 2013). On the other hand, these new genes are also known to have a fast turnover rate: Estimates based on synonymous substitution rates showed that the majority of duplicated genes are silenced after 4 My in eukaryotes (Lynch and Conery 2000, 2003). Moreover, comparative genomics across closely related species demonstrated that the X chromosome was a hot bed in recruiting duplicated and de novo new genes in both mammals and *Drosophila* that appeared to be lost rapidly soon after a short period (Zhang et al. 2010a, b). It is thus possible that LoF mutations might be enriched in new genes, especially X-linked ones, resulting in the loss of these genes. To further support this idea, we note that new genes share two features with those genes containing LoF variants: Limited tissue transcription and possession of at least one paralog, except in the case of de novo genes (Kaessmann 2010; Zhang et al. 2012). These trends naturally raise the question of how much these two groups overlap and what this overlap means for the gain and loss of new genes.

Here, we utilized the resequencing data from 162 strains in the *Drosophila* Genetic Reference Panel (DGRP) (Mackay et al. 2012) to investigate the frequency distribution of LoF mutations at the population level. There are various types of LoF variants, such as PTCs, loss of start codon, frame-disrupting indels, and large deletions. In this study, we focus on PTCs because calling indels and structural variations based on short-read sequencing is still problematic (Handsaker et al. 2011). Moreover, PTCs are the most abundant single nucleotide polymorphism (SNP)-mediated variants of gene loss (MacArthur et al. 2012). Combined with our previously generated whole-genome gene age data (Zhang et al. 2010a), we characterized PTC-encoding genes in terms of their functions (approximated by their transcriptional profile) and evolutionary age across different PTC frequency intervals.

We found interesting patterns for genes harboring different frequency PTCs. On one hand, genes encoding either low-, moderate-, or high-frequency PTCs are all narrowly transcribed compared with the genomic background, while the propensity gets stronger with the increase of allele frequency. Moderate-frequency PTCs are enriched in genes expressed highly in the midgut, whereas genes encoding high-frequency PTCs tend to be expressed or uniquely in reproductive organs. On the other hand, at least 16% of genes harboring low-frequency PTCs, 30% of genes harboring moderate-frequency PTCs, and 50% of genes harboring high-frequency PTCs are evolutionarily young genes, defined as those that emerged in the last 60 My. Thus, expression profile and evolutionary age jointly affect the probability of occurrence and frequency of PTC variants in *Drosophila*. Moreover, such a result also modifies the previous view on new gene evolution, suggesting that following a period of rapid gene loss after fixation (Lynch and Conery 2000), there could be a prolonged period of gene loss.

## Results

### Approximately 1,000 PTCs Were Identified in the DGRP Population, Which Were Mostly Genuine

We developed a bioinformatics pipeline (Materials and Methods) to identify putative PTCs based on the pseudochromosome assembly data from the DGRP (Mackay et al. 2012). To be conservative, we required the putative PTCs to disrupt all annotated isoforms of the host gene, that is, they had to be constitutive. We also required that a putative PTC removes at least 5% of the coding region in each isoform (MacArthur et al. 2012). To distinguish gene loss from gene gain, we inferred the derived state for each PTC-encoding polymorphic site using syntenic genomic alignment between *Drosophila melanogaster* (*D. mel*), *D. simulans* (*D. sim*), and *D. yakuba* (*D. yak*) (Materials and Methods). In total, we identified 972 putative PTC SNPs distributed across 819 genes (supplementary table S1, Supplementary Material online).

Sequencing errors could cause misidentification of PTCs (Lee and Reinhardt 2012; MacArthur et al. 2012). To investigate the extent of this issue, we conducted polymerase chain reaction (PCR) and Sanger sequencing for 94 PTC sites randomly chosen to cover the allele frequency spectrum. For each site, we randomly selected and sequenced one PTC-encoding strain and one contra-PTC strain (a strain with the functional allele). From these sequences, we estimated the true positive rates (TPR) for PTC and contra-PTC sites to be 87% (75/86 validated) and 100% (87/87 validated), respectively (supplementary table S2, Supplementary Material online). The remaining eight PTC and seven contra-PTC validation experiments failed at either the PCR or sequencing steps.

Notably, the false positive rates (FPR) appear to be different for low-, moderate-, and high-frequency PTCs—20% (6 out of 30), 13% (5 out of 38), and 0% (0 out of 18), respectively (supplementary table S2, Supplementary Material online). Further inspection of the sequencing results revealed that all five “false positives” in the moderate-frequency group were due to one particular strain—RAL786 (supplementary table S2, Supplementary Material online). In other words, all sequencing validations involving this strain failed to match the genome sequence of the strain, leading us to suspect that it was mislabeled. If we remove this strain, both high- and moderate-frequency groups would reach 100% validation rate based on 18 and 33 data points, respectively. In contrast, four out of 28 in the low-frequency group were false positives, with all four being singletons (supplementary table S2, Supplementary Material online). Such a result clearly suggests that singletons are uniquely prone to errors, as expected if SNP calling in different strains is independent. A similar correlation between minor allele frequency and FPR has previously been reported (Guo et al. 2013). Herein, to be conservative, we still keep the validation result of strain RAL786 and consider the FPR for three groups as 20%, 13%, and 0%, respectively. Differences in error rates between allele frequency groups may confound downstream analyses. To account for this issue, we generated 100 pseudo data sets by randomly removing putative PTCs according to the estimated FPR for each allele frequency group. We used these pseudo data sets to validate the major conclusions (Materials and Methods). We found that all results we reported below were unaffected by the sampling procedure. For brevity, we present the unsampled results in the main text, and we included representative results using sampled data in the supplementary materials, Supplementary Material online.

As a second approach to validate the putative PTC sites, we tested the prediction that the PTC-encoding alleles should be expressed lower than the functional alleles, due to the possibility of PTCs producing a truncated open reading frame, which in turn leads to the degradation of mRNA via the nonsense-mediated decay process (Chang et al. 2007). However, it should be noted that this prediction is not expected to be true for all PTCs. For example, a significant reduction in gene expression was found for only 16.3% of LoF-encoding genes in humans (MacArthur et al. 2012). By analyzing the whole-body transcriptome data available for the 40 core DGRP strains (Materials and Methods), we found that 25.5% of our PTC alleles exhibited significantly lower expression than the functional (ancestral) alleles, while only 0.8% displayed the opposite expression pattern. For comparison, we randomly chose a set of nonsynonymous (missense) or synonymous polymorphisms matching the derived allele frequency (DAF) of the PTC variants. Among these sites, we found that the alleles containing the derived mutations are also more likely to be expressed lower than the ancestral alleles, with the difference being more pronounced for nonsynonymous mutations. However, the extent of allele imbalance for these two classes of mutations is smaller compared with PTC polymorphisms (0.6% vs*.* 0.2% for nonsynonymous mutations and 0.4% vs. 0.2% for synonymous mutations, respectively; table 1). In conclusion, we found that alleles harboring derived PTC mutations are much more likely than alleles containing derived synonymous or missense mutations to have a lower expression level compared with the ancestral alleles.

**Table 1.** Comparison of Expression Levels between Derived and Ancestral Alleles for Different Functional Classes.

Gene Category	Total	Lower in Derived Allele	Lower in Ancestral Allele
Genes w/ PTC Polymorphism	239	61 (25.5%)	2 (0.8%)
Genes w/ Nonsynonymous Polymorphism	2,390	15 (0.6%)	5 (0.2%)
Genes w/ Synonymous Polymorphism	2,390	10 (0.4%)	5 (0.2%)

Note.—The DGRP generated whole-body microarray expression data for the 40 core DGRP strains, which covered 239 PTCs distributed across 210 genes. In these 40 strains, we randomly sampled 2,390 nonsynonymous sites and synonymous sites by controlling their allelic frequency according to PTCs’. For each gene of interest, we compared strains encoding PTC variants and those harboring wild-type alleles with the Wilcoxon rank test followed by multiple testing correction (Materials and Methods). Only tests with FDR lower than 0.05 were counted. For example, for 15 sites out of the 2,390 nonsynonymous sites, the derived alleles had significantly lower expression levels compared with the ancestral alleles.

We finally examined the DAF spectrum of PTC SNPs, with the expectation that true PTC variants should be strongly biased toward the low-frequency end. Consistent with the previous genome-wide survey of LoF mutations (Lee and Reinhardt 2012; MacArthur et al. 2012), the frequency spectrum for our putative PTCs is strongly skewed toward the low-frequency end compared with both synonymous and nonsynonymous SNPs (supplementary fig. S1, Supplementary Material online). This result suggests that these PTCs are under intense purifying selection. The extent of the skew we observed in the DGRP is much stronger than that previously reported in humans: A majority (86%) of the putative PTCs we identified have an allele frequency under 2%, compared with 55% of human PTCs in the same frequency range (MacArthur et al. 2012). This is consistent with the fact that *D. mel* has a much larger population size than human; hence, selection in *D. mel* is more efficient (Gillespie 2004; Cutter and Payseur 2013).

Therefore, all three lines of evidence indicate that the majority of PTC SNPs in our data set are genuine LoF mutations. Based on such a high-quality data set, we further investigated how PTC-encoding genes with different allele frequencies differ in their transcriptional profiles in different tissues.

### PTC-Encoding Genes Have Distinct Tissue-Biased Expression That Depends on the PTC Allele Frequency

**Fig. 1.**
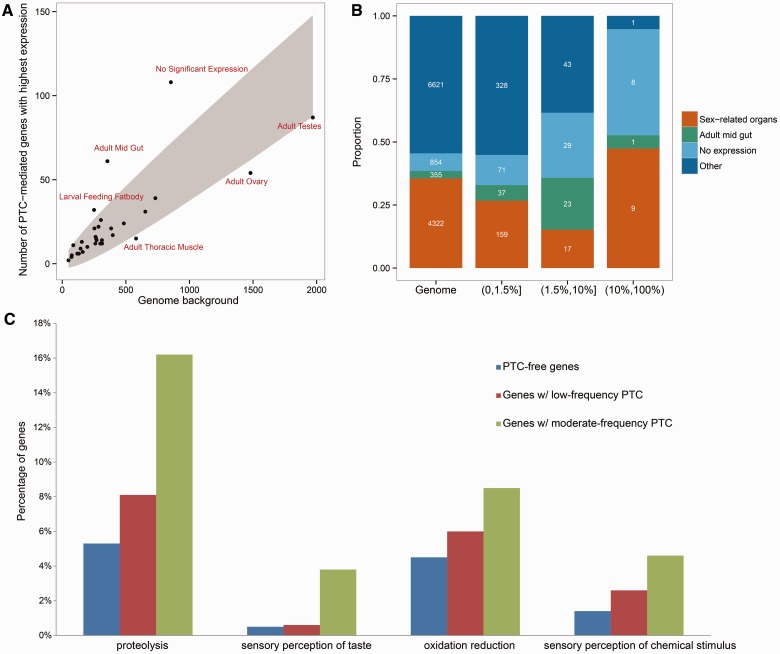
Expression profile of PTC-encoding genes. (*A*) Scatter plot comparing the distributions of genes either genome-wide (*x* axis) or among PTC-encoding genes (*y* axis), grouped by the tissue types in which they are most highly expressed (based on the FlyAtlas data). The 30 tissue types included in FlyAtlas are plotted. The gray ribbon represents the 95% confidence interval (5% family wide type-I error rate). We assumed a multinomial distribution for the number of PTC genes in each tissue. The center of the ribbon is the predicted number of PTC-encoding genes based on the genome-wide frequency. (*B*) Detailed comparisons for PTC-encoding genes grouped by the PTC variant DAFs. The 30 tissue types were separated into four groups. The sex-related tissue group includes six tissue types in the original set (see highlighted terms in supplementary table S3, Supplementary Material online). (*C*) Gene ontology (GO) enrichment for genes harboring PTCs. The proportion of each functional class was obtained from DAVID (http://david.abcc.ncifcrf.gov), and only the top four GO terms for the genes with moderate-frequency PTCs are shown. Genes with high-frequency PTCs have no significant GO terms due to the small sample size and are therefore not shown. Low, moderate, and high denote a DAF ≤1.5%, 1.5–10%, and >10%, respectively.

We first examined the tissue-biased expression pattern for PTC-encoding genes in *Drosophila*, using the microarray-based tissue profiling data in FlyAtlas (Chintapalli et al. 2007). For each PTC-encoding gene, we recorded its top expressing tissue type. We then counted the number of genes in each tissue type and compared their distribution to the distribution for all genes (fig. 1*A*). The results revealed two unexpected patterns: 1) the adult midgut is strongly enriched for PTC-encoding genes; and 2) there is a significant excess of PTC-encoding genes with no detectable expression across all 30 tissue types.

To further investigate whether genes encoding PTC variants of different DAFs exhibit distinct tissue preferences, we grouped PTC-encoding genes into three categories, low PTC frequency (0–1.5%), moderate PTC frequency (1.5–10%), and high PTC frequency (10–100%). The cutoffs are based on the following two reasons: 1) the tissue expression profile show dramatic changes across these thresholds (supplementary fig. S2, Supplementary Material online); and 2) this set of cutoffs ensures a relatively large sample size for each group. Comparison of the “highest-expression tissue” profile between the three PTC frequency groups and the genome background revealed both shared and distinct patterns (fig. 1*B*). Nonexpressed genes (i.e., “No expression” in fig. 1*B*) are significantly enriched in PTC-encoding genes for all three groups (11.9%, 25.9%, and 42.1% vs. 7% for low-, moderate-, and high-frequency groups versus genome-wide background, Fisher’s Exact Test or FET *P* < 0.001, supplementary table S3, Supplementary Material online). In contrast, the enrichment of genes most highly expressed in the midgut is mainly attributable to the low- and moderate-DAF groups: 6% (37/595) of the low-DAF group and 20.5% (23/112) of the moderate-DAF group have the highest expression in the adult midgut, compared with 3% of the genome-wide background (FET *P* = 4.4 × 10^−^^5^ and 3.4 × 10^−^^13^, respectively, fig. 1*B*, supplementary fig. S3*A* and table S3, Supplementary Material online). Finally, within the high-frequency DAF group consisting of 19 genes, nine out of the 11 genes with detectable expression in FlyAtlas show the highest expression in sex-related tissues, including one gene most highly expressed in the male ejaculatory duct, two genes in the accessory gland, two genes in the testis, and four genes in the ovaries (supplementary fig. S3*B* and table S3, Supplementary Material online). If we only consider genes showing detectable expression in at least one tissue type, 82% (9 out of 11) of these genes have their highest expression in sex-related tissues, which is significantly higher than the genome-wide level of 38% (FET *P* = 0.01).

Our measure for the top expression tissue has the potential to include genes that are broadly expressed rather than tissue-specific. To explore this issue, we defined a data set of tissue-specific genes by specifying the tissue specificity index (*τ*) cutoff of greater than 0.85 (Materials and Methods) (Yanai et al. 2004) and examined how these genes were distributed with the increase in DAFs of PTCs. As shown in supplementary figure S4, Supplementary Material online, the midgut again stands out as the top enriched tissue for the group harboring moderate-frequency PTCs (42% vs. 10%, FET *P* = 5.5 × 10^−^^8^). Meanwhile, the high-DAF group is dominated by sex-biased genes (six out of seven genes are only expressed in sex-related organs), although the difference is not significant in this small sample (86% vs. 57%, FET *P* > 0.05).

The overall pattern was also robust when we used RNA-seq data generated by modENCODE (Graveley et al. 2011) as compared with FlyAtlas (supplementary figs. S5 and S6, Supplementary Material online). In short, genes harboring moderate-frequency PTCs are enriched for the highest expression in the midgut, which is the primary digestion organ in insects (Pridgeon et al. 2003); genes harboring high-frequency PTCs are enriched in sex-related organs. The functional role of the moderate-DAF group of PTC-encoding genes in the adult midgut is also supported by Gene Ontology terms, that is, proteolysis and oxidative reduction, which are consistent with the digestive function of the organ (fig. 1*C*). On the other hand, genes with high-frequency PTCs tend to be sex-biased, which was also confirmed with the Sebida database (supplementary table S4, Supplementary Material online) (Gnad and Parsch 2006).

To explain the enrichment of genes with no detectable expression across all 30 tissues, we hypothesize that some of these genes are induced by environmental stress or other stimuli and are thus not captured in the steady-state measurements by FlyAtlas. One supporting example is *Victoria* or *CG33117*, which is mainly activated under stresses such as bacterial infection and heat shock (Ekengren et al. 2001).

Interestingly, genes harboring moderate-frequency PTCs may be also related with environmental change and thus implicated in immune response. Specifically, genes expressed most highly in the midgut, which dominates the moderate-frequency PTC group, are either directly or indirectly regulated by *ATF3*, the master regulator of metabolic and immune system homeostasis (Rynes et al. 2012). Conversely, when *ATF3* was disrupted, as many as 15% of the genes in the moderate-DAF category showed significant changes in expression compared with 6% of the PTC-free genes and 10% of the PTC-encoding genes with low-DAF PTC variants, whereas none of the high-DAF genes showed a significant change (FET *P* < 0.001; Materials and Methods).

### PTC-Encoding Genes Are More Narrowly Transcribed and Show Distinct Chromosome Distribution Patterns

**Fig. 2.**
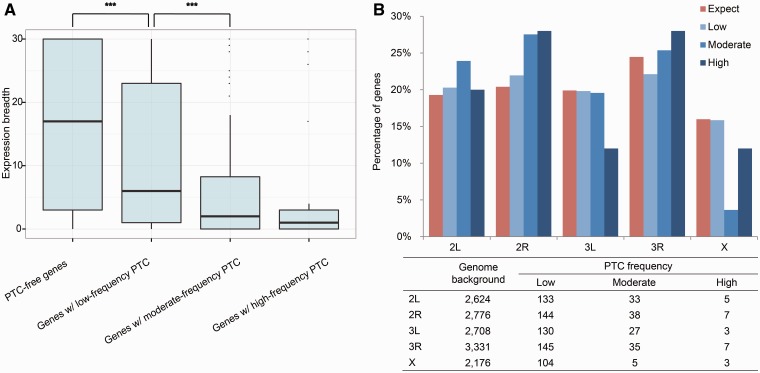
Differences among the three frequency groups in expression breadth and chromosomal distribution. (*A*) Distribution of the expression breadth of PTC-free genes and genes with PTCs of different frequencies. Expression breadth is measured as the number of tissues in which the focal gene is present (Materials and Methods). *** denotes *P* < 0.001 with the Wilcoxon rank test. (*B*) Chromosomal distribution of genes with PTCs in different frequency groups. The table below shows the counts underlying this graph. Low, moderate, and high denote DAFs ≤1.5%, 1.5–10%, and >10%, respectively.

Previous studies showed that PTC-encoding genes tend to have more tissue-specific expression patterns (Lee and Reinhardt 2012; MacArthur et al. 2012). To test this hypothesis, we measured the breadth of expression for both PTC-encoding and PTC-free genes by counting the total number of tissues in which expression of the gene was detected beyond the background level (Materials and Methods). As shown in figure 2*A*, PTC-free genes were detected, on average, in 17 tissues. In contrast, the average number for low-, moderate-, and high-frequency PTC genes was six, two, and one tissue(s). Analogously, *τ* also increases with the PTC allele frequency (supplementary fig. S7, Supplementary Material online). One possible explanation is that genes with narrower or specific transcription profiles are less likely to produce detrimental effects when PTC occurs.

Additionally, we found that genes with moderate-frequency PTCs are significantly underrepresented on the X chromosome compared with the genome background (FET *P* = 9.7 × 10^−^^6^, fig. 2*B*), whereas the other two categories are evenly distributed (FET *P* > 0.1, fig. 2*B*). As reported previously (Zhang et al. 2010a; Mikhaylova and Nurminsky 2011; Vibranovski et al. 2012), the X chromosome overall is depleted of somatic tissue-biased genes, such as midgut-biased genes (supplementary table S5, Supplementary Material online), but enriched for evolutionarily old ovary-biased and new testis-biased genes (Zhang et al. 2010a, b). Such a gene content bias contributes to the paucity of genes with moderate-frequency PTCs on the X chromosome.

### PTC-Encoding Genes Are Enriched for Evolutionarily Young Genes

Given that both new genes (Zhang et al. 2010a) and genes harboring high-frequency PTCs (fig. 1*B*) tend to show testis-biased or testis-specific expression, we asked whether these two groups significantly overlapped. Using previously generated age data in *D. mel* (Zhang et al. 2010a), we defined “young genes” as those that originated after the split of the subgenera *Sophophora* and *Drosophila*. We found that these young genes are more likely to contain PTCs compared with old genes predating the split (14% vs*.* 6%, FET *P* = 2.2 × 10^−^^16^; table 2). Moreover, the proportion of young genes increases with the DAF of the PTC variants: Genes that contain moderate- and high-frequency PTCs have a significantly higher proportion of young genes than those with low-frequency PTCs (30% and 50% vs. 16%, FET *P* = 0.001 and 0.004, respectively; table 2). After manually curating genes that were not dated in the previous automatic analysis (Zhang et al. 2010a) (supplementary table S6, Supplementary Material online), the trend remained robust (39% and 67% vs*.* 22%; supplementary fig. S8, Supplementary Material online; Materials and Methods). These results suggest that PTC may make an important contribution to the fast turnover of new genes within a species, extending the previous analysis that showed a similar pattern in between-species comparisons (Lynch and Conery 2000; Zhang et al. 2010a, b).

**Table 2.** Comparison of PTC Frequency Spectra between Young and Old Genes and among Young Genes with Different Origination Mechanisms.

Gene Category	PTC-Free	PTC Frequency		
		Low	Moderate	High
Old Gene	10,580	460	74	7
Young Gene	764	89	32	7
Percentage of Young Gene	7%	16%	30%	50%
DNA-Based Young Gene	329 (589)	42 (77)	18 (30)	3 (5)
RNA-Based Young Gene	76 (91)	6 (10)	0 (0)	0 (0)
De Novo Gene	84 (84)	2 (2)	2 (2)	2 (2)

Note.—The numbers inside the parentheses are the raw counts, whereas the numbers outside the parentheses are the gene counts with a more strict parent-child relationship (i.e., the parent gene must be older than the child gene). These numbers were used for statistical tests.

New genes originated through different mechanisms, which may affect their probability of accumulating PTCs. For example, we expect that genes that originated through DNA-based duplication and retroposition are more likely to contain PTCs compared with de novo genes, because in the former but not the latter case, the existing paralogs could compensate for their loss of function. To test this hypothesis, we compared the proportion of PTC-encoding genes between the three classes of young genes. We found that DNA-based duplicated genes are indeed more likely to encode PTCs compared with de novo genes (16% *vs.* 7%, one side FET *P* = 0.01; table 2). In contrast, RNA-based duplicated genes (also known as retrogenes) have the same likelihood as de novo genes to encode PTCs (7% vs. 7%, FET *P* = 1). We further focused on the proportion of young genes encoding moderate- and high-frequency PTCs because these two classes are more likely to lead to PTC-mediated gene loss. Interestingly, we found that although retrogenes share the same overall probability of encoding PTCs as de novo genes, they were the least tolerant of PTCs of moderate- or high-frequency—none of the 82 candidate retrogenes encoded moderate- or high-frequency PTCs. In comparison, 21 out of the 392 DNA-based duplicates and four of the 90 de novo genes did encode moderate- or high-frequency PTCs (compared with retrogenes, one side FET *P* = 0.02 and 0.07, respectively; table 2). This result was robust when we used a less stringent method to categorize new genes by their origination mechanisms (table 2). In conclusion, we found that DNA-based duplicates are much more likely to accumulate PTCs as predicted by the paralog hypothesis. Unexpectedly, RNA-based duplicates appear to be the least tolerant of moderate- or high-frequency PTCs. Finally, even though de novo genes do not have compensating paralogs, they are still occasionally subject to PTC-mediated gene loss.

**Fig. 3.**
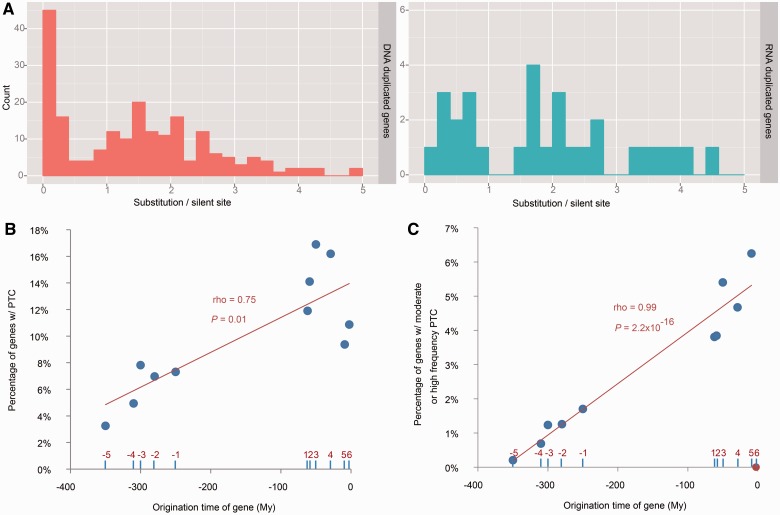
Rapid turnover of young genes and PTC-mediated gene loss. (*A*) Distribution of synonymous substitutions per site between parent and child copies for either DNA-based (left) or RNA-based (right) duplicates; (*B*) Percentage of PTC-encoding genes in each age group; (*C*) Percentage of genes harboring moderate- or high-frequency PTCs in each age group. Branches 1–6 followed previous results (Zhang et al. 2010a); older branches −5 to −1 were inferred based on more distantly related insect species (Materials and Methods). The *x* axis represents the middle point for each branch in million years. For instance, *D. mel* specific genes assigned to branch 6 has an *x* value of −2.5 My, indicating that the origination time for this group ranged from −5 to 0 My. The evolutionary time for the oldest age group was arbitrarily set as −350 My. A linear regression line is shown in Panel *B* (all points) and Panel *C* (the youngest group is excluded and marked in red). The split times of different species are derived from former studies (Tamura et al. 2004; Hedges et al. 2006).

Moreover, table 2 also indicates that the previously observed rapid loss of duplicated genes (Lynch and Conery 2000) is mainly attributable to DNA-based duplicates, which have much higher probabilities of encoding moderate- or high-frequency PTCs. A direct way to test this hypothesis is to repeat a previous similar analysis (Lynch and Conery 2000). Briefly, they used synonymous divergence between a pair of duplicates (denoted as *S*) as a proxy for their age, assuming that silent changes accumulate over time at a constant (neutral) rate. The authors found that the highest proportion of duplicates is in the low *S* group, with the frequency going down as *S* increases (Lynch and Conery 2000). This pattern was used to suggest a high rate of birth followed by a rapid death model for gene duplication. We found that this pattern of overrepresentation in low divergence groups is only observed among DNA-based duplicates but not RNA-based duplicates (fig. 3*A*). We therefore propose a refined model in which only DNA-based duplicates but not RNA-based duplicates are subject to the high rate of birth followed by rapid death.

To take a closer look at the correlation between gene age and PTC-encoding probability, we further divided young genes into six age groups as conducted previously (Zhang et al. 2010a). We partitioned old genes predating the genus *Drosophila* split into five age groups by integrating the genomic data from several insects such as mosquito and honeybee (Materials and Methods). As expected, the proportion of genes harboring PTCs decreased gradually with the increase of gene age (fig. 3*B*), roughly following a linear curve (Spearman’s rho = 0.75, *P* = 0.01). We further reasoned that low-frequency PTCs are expected to contain a high proportion of deleterious mutations, which are unlikely to be fixed in the population in the future. Therefore, we reanalyzed the data using only genes harboring moderate- and high-frequency PTCs and found a strong linear correlation except for one data point (fig. 3*C*). This outlier point corresponds to the *D. mel*-specific genes, that is, genes that have emerged in the last 2.5 My. This group of genes contains no moderate- or high-frequency PTCs. There may be two reasons for this result. First, recently derived duplicates, due to their high similarity to the parent copies, cause problems in read mapping—nonuniquely mapped short reads are generally discarded, leaving undetermined sites (“N”) in their positions. For 74% of genes in the youngest group, we found that at least 30% of the coding sequences (CDS) are marked as N. This severe missing data problem is specific to the youngest group because this proportion drops below 19% for the second youngest age group, and it continues to decrease in the other groups (supplementary table S7, Supplementary Material online). Additionally, high similarity between parent and child copies also makes the youngest age group more susceptible to gene conversion events, which exacerbates the mapping problem (Osada and Innan 2008) (supplementary fig. S9 and table S7, Supplementary Material online). Second, we hypothesize that the youngest group of genes has not had enough time for new mutations to rise to moderate or high frequencies. To test this hypothesis, we examined the DAF spectrum for synonymous sites among the different age groups. If our hypothesis were correct, we would expect to see the lowest DAF for synonymous SNPs in the youngest gene group, which is indeed observed in supplementary figure S10, Supplementary Material online. Wilcoxon rank test is significant for all comparisons between the youngest group and all the other groups (*P* = 1.5 × 10^−^^8^ ∼2.1 × 10^−^^3^), except for the second youngest one which is possibly due to the small sample size. Therefore, we concluded that the youngest group of genes is an outlier in terms of PTC allele frequency distribution. With this data point excluded, we found that the remaining points have a near-perfect linear relationship between the time since origination of the gene and the proportion of genes harboring moderate- and high-frequency PTCs (Spearman’s rho = 0.99, *P* = 2.2 × 10^−^^16^). Notably, only 0.3% of the oldest genes (Branch -5, fig. 3*C*) shared by various insect lineages encode such PTCs, which is significantly less than all the other age groups besides *D. mel* specific genes (1–5%, FET *P* ≤ 0.05). Genes shared between *D. mel* and *D. sim*, that is, genes that emerged between 5 and 13 Ma, contain the highest proportion of moderate- and high-frequency PTCs—more than 6% of genes harbor such mutations, suggesting that these genes are on the way being lost. This pattern is in concordance with the result of a previous study showing that most duplicated genes disappear 4 My after their origination (Lynch and Conery 2003).

## Discussion

In the DGRP population, we studied one type of LoF, PTCs, with respect to their DAFs, transcriptional profiles, chromosomal distributions and roles in the loss of new genes. We confirmed the previous finding that PTC-containing genes tend to have narrow expression breath (Lee and Reinhardt 2012; MacArthur et al. 2012). Furthermore, we also discovered two novel patterns. First, different DAF groups show distinct tissue specificities: Genes harboring moderate-frequency PTCs are enriched for midgut expression, whereas those with high-frequency PTCs are almost exclusively expressed in sex-related organs. Second, genes harboring PTCs tend to be evolutionarily young and this trend is more pronounced in moderate- and high-frequency PTC groups. Different origination mechanisms such as DNA-based, RNA-based, or de novo origination are associated with different PTC allele frequency distributions, with RNA-based duplicates showing the lowest probability of encoding moderate- and high-frequency PTCs. These two findings shed new light on PTC-mediated gene loss in fruit flies.

### Pleiotropy, As Approximated by Expression Profile and Gene Age, Shapes the Landscape of PTC-Mediated Gene Loss

It has been argued that selection tends to minimize the negative consequences of pleiotropy (Carroll 2005) and that genes involved in fewer biological processes are less pleiotropic (He and Zhang 2006). Therefore, genes of low pleiotropy are more likely to be lost. Our analyses revealed that genes with narrower expression profiles and those that emerged in the recent evolutionary period are more likely to contain nonsense mutations. We hypothesize that with all other factors being equal, genes with limited expression breadth (in fewer tissues) should be involved in fewer processes, which means they are less pleiotropic and more likely to accumulate PTCs. This hypothesis is supported by our finding (fig. 2*A*). Additionally, we observed that genes with predominant or exclusive expression in organs interacting with the environment (e.g., midgut) or organs under intense selection pressure (e.g., sex-related organs) are more likely to be subject to rapid turnover (fig. 1). This result suggests that while negative selection limits PTC distribution to low pleiotropy genes, positive selection pressure may favor certain functional categories for evolutionary turnover. Additionally, gustatory receptor genes, which are involved in sensory perception, are also enriched in the moderate-PTC group (fig. 1*C* and supplementary table S8, Supplementary Material online), which again supports our hypothesis that genes involved in interacting with the environment are more prone to PTC-mediated gene loss.

Using expression breadth alone as a proxy for pleiotropic effects of a gene is certainly inadequate. Indeed, pleiotropy can manifest on many levels, of which expression breadth is just one dimension. Furthermore, even if we only consider expression breadth, we rely on tissue profiling databases such as FlyAtlas or modENCODE, which do not exhaust all possible developmental stages or conditions and thus only provide a partial view. To overcome this challenge, we suggest using gene age as a second proxy for pleiotropy. The logic behind this decision is 2-fold: First, there is a positive correlation between gene age and the expression breadth as well as intensity, such that the former can serve as a surrogate for the latter (Zhang et al. 2012; Almada et al. 2013); second, pleiotropy is known to be positively correlated with sequence conservation (He and Zhang 2006), which is also positively correlated with gene age by definition. Thus, young genes are less conserved and expected to have less pleiotropy compared with old genes.

In addition to the overall higher proportion of younger genes harboring PTCs compared with older genes (table 2 and fig. 3), three other findings further illustrate the importance of gene age in determining the propensity of gene loss. First, for DNA-based duplicates, the child or the younger copy is more likely to contain PTCs than the parent copy: Among the 392 gene pairs, 63 child genes harbor PTCs, while only 33 parent genes do (16% vs. 9%, FET *P* = 0.003, supplementary table S9, Supplementary Material online). Second, young de novo genes are also more likely to harbor moderate- and high-frequency PTCs compared with old singleton genes (4.4% vs. 0.7%, FET *P* < 0.005; Materials and Methods). Last and most importantly, young genes are more likely to encode PTCs compared with old genes, even after controlling for their expression breadth; for those genes present only in one or zero tissues profiled by FlyAtlas, young ones are still more likely to harbor PTCs compared with old ones (14% vs. 8%, FET *P* = 0.002, supplementary table S10, Supplementary Material online). In other words, the strong negative correlation between gene age and the proportion of segregating PTCs (fig. 3*B* and *C*) occurs not merely because new genes tend to be narrowly transcribed (Zhang et al. 2012). In conclusion, we suggest that pleiotropy, as approximated jointly by expression profile and gene age, shapes the landscape of PTC-mediated gene loss.

### Non-retroposed Young Genes Go through a Long Process from Fixation to Preservation

Our observation that evolutionarily young genes are overrepresented among PTC-encoding genes is consistent with previous indications of rapid turnover of new genes based on between-species analyses (Lynch and Conery 2000; Zhang et al. 2010a, b). However, we were still surprised by the extent of the enrichment. Specifically, the young genes that originated after the genus *Drosophila* split or those genes that emerged in the last 60 My account for at least one-third of the genes harboring moderate-frequency PTCs and half of the genes harboring high-frequency PTCs (table 2), even though this group of genes accounts for a mere 9% of the whole genome.

**Fig. 4.**
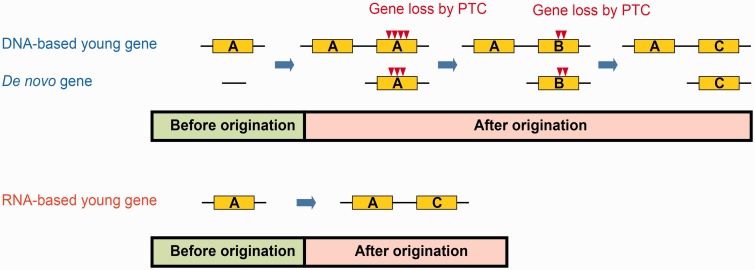
Evolutionary fate of new genes. (*A*) LoF mutations such as PTCs can accumulate in DNA-based young genes and de novo genes after their origination. The single-copy gene A is duplicated or a *de novo* new gene emerges. In the direction indicated by the blue arrow, the derived copy either gains LoF mutations (red arrows) or is finally preserved or diverged as C. The different densities of the red arrows indicate that LoF alleles occur more frequently for genes with younger ages and less frequently for those with older age. (*B*) RNA-based duplicated genes reach the final preservation phase much faster, and thus, they could not tolerate PTCs. The parent gene A is retroduplicated as C, which is preserved.

Classic evolutionary theory divided the evolutionary path of duplicated new genes into the pre-fixation, fixation, fate-determination, and preservation phases (Innan and Kondrashov 2010). The pattern in figure 3*B* indicates that new genes, even after becoming fixed in the population, may still face a very high probability of being lost in the fate-determination phase. Furthermore, our data suggest that this latter phase could be far longer than previously thought—even genes that emerged 250 Ma or longer have an appreciable chance of being lost, as indicated by the proportion of genes encoding moderate- to high-frequency PTCs. One caveat of our analysis is that we may have underestimated the age for old yet fast-evolving genes, which, in our homology-based classification scheme, are likely to be misclassified into younger groups (Elhaik et al. 2006). Because these genes are less pleiotropic and may be more likely to encode PTCs (He and Zhang 2006), this will lead to an increase in the proportion of PTC-encoding genes in the younger gene groups. However, this issue should least affect the younger age group (1–6 in fig. 3) because genome alignment-based synteny together with closely related species comparison within the *Drosophila* genus increases the chance of correct ortholog identification (Zhang et al. 2010a). Thus, the curve for the last 60 My (1–6 age groups in fig. 3) should be more reliable than that for the older groups; the fate-determination stage could still be as long as 60 My. This conservative estimate is still much longer than the half-life of 4 My of duplicated genes described previously (Lynch and Conery 2003). Thus, our results suggest a revision of the classic model, in which the fate-determination phase is appreciably long and the probability of gene loss steadily declines when a gene gets older (fig. 3*C*). Moreover, genes of de novo origin follow the same model. Thus, we updated the previous model (Innan and Kondrashov 2010) as shown in figure 4*A*, the main feature of which is a long period from fixation to preservation.

Interestingly, retrogenes appear to be resistant to loss, with none of the 82 cases encoding moderate- or high-frequency PTCs (table 2), suggesting a very short fate-determination phase or that the fixation and fate-determination phases may be overlapping (fig. 4*B*). It was reported that retrogenes lose their original regulatory elements but maintain protein-level functionality (Brosius 2003; Katju 2012). Compared with DNA-based duplicates, retrogenes are more likely to undergo neofunctionalization and play radically different functions (Katju 2012). In fact, recent work via expression profiling demonstrated that nearly all retrogenes were retained by neofunctionalization in *Drosophila* (Assis and Bachtrog 2013). Therefore, the small functional redundancy between a retrogene and its parent gene suggests that either one can seldom compensate for the loss of the other. In contrast, DNA-based duplicates share more functional similarity with the corresponding parent genes, and thus may be more likely to accumulate LoF variants. However, compared with de novo genes with short newborn peptides (supplementary fig. S11, Supplementary Material online), the proteins of retrogenes are capable of playing more diverse functions (Siepel 2009; Carvunis et al. 2012). That is, the protein-level pleiotropy of retrogenes will be generally higher than that of de novo genes, and therefore, retrogenes would be less likely to be lost.

### The Loss of X-Linked New Genes May Be Driven by Frame-Disrupting Mutations or Large Deletions

Patterns of within population polymorphisms should resemble those of between species substitutions, should the same evolutionary forces be acting in both short and long time-scales. Because of this reason, we expect X-linked genes to be more likely to contain PTC polymorphisms, especially those moderate- or high-frequency PTCs, because X-linked genes were known to be subject to higher rates of gene gain and loss compared with autosomal ones (Zhang et al. 2010a,b) and PTCs with relatively higher frequency are more likely to lead to fixed loss event. However, we failed to find a higher rate of X-linked moderate- or high-frequency PTCs compared with the autosomal counterpart (fig. 2*B*). In fact, for moderate-frequency PTCs, we observed the reverse trend, that is, X-linked genes are underrepresented in that group. Such a complex pattern could be jointly explained by the difference of selection efficiency and gene content bias. On one hand, PTCs are most likely to be recessive deleterious as discussed before (Lee and Reinhardt 2012). Thus, such sites would be under stronger purifying selection if linked with X chromosome because their deleterious effects would become dominant in males (Vicoso and Charlesworth 2006), which leads to their depletion on the X. On the other hand, gene content is not uniformly distributed across different allele frequency groups and different chromosomes. Specifically, the moderate-frequency PTC group is highly enriched for midgut-expressed genes, which are actually underrepresented on the X chromosome (supplementary table S5, Supplementary Material online, 11.3% X-linked vs. 16.1% X-linked among all genes; FET *P* = 0.03). In contrast, the high-frequency PTC group is overrepresented with sex-related genes, whereas X chromosome is known to enrich old ovary-expressed genes and new testis-expressed genes (Zhang et al. 2010a). With these two aspects, the depletion of moderate-frequency PTCs on X chromosome could be caused by both their recessive deleterious nature and the underrepresentation of midgut-expressed genes. Analogously, the even distribution of genes harboring high-frequency PTCs could be because that high-frequency PTCs are less likely to be recessive deleterious than moderate-frequency PTCs or because that sex-related genes are overall not depleted from X chromosome.

To test whether the loss of X-linked genes was driven by other types of LoF mutations, we also identified 112 and 243 substitutions disrupting start codons and splice sites, respectively (supplementary tables S11 and S12, Supplementary Material online). Genes encoding these mutations with similarly high frequency (≤10%) were not overrepresented in X chromosome either (supplementary fig. S12, Supplementary Material online). Another possibility is that the loss of X-linked new genes is mainly driven by yet uncharacterized types of variations, for example, frame-disrupting mutations or large deletions. With the increase in read length and accuracy offered by next- and third-generation sequencing, such possibilities can be tested.

## Materials and Methods

### Sequencing Data Analysis

Genomic sequences of the 162 strains were obtained in the format of pseudochromosomes from the DGRP (freeze1) (Ayroles et al. 2009; Mackay et al. 2012). The annotation file of *D. mel* (BDGP5.25.65) was downloaded from Ensembl (http://www.ensembl.org) (Flicek et al. 2012). Genes were removed from the annotation file if any of the following errors occurred in any isoform: 1) the length of an annotated CDS was not a multiple of three; 2) a premature termination codon was present based on the reference genome; 3) the start and stop codons of the CDS were not ATG and TAA, TAG, or TGA, respectively. After this filtering step, 13,615 protein-coding genes were retained for further analysis. We focused on SNPs. Moreover, only biallelic polymorphisms were included in our analysis. PTCs, synonymous, and nonsynonymous SNPs were identified using in-house Perl code and the reference genome. Splicing site mutations were obtained through base pair-level comparisons at the boundaries of introns. Furthermore, we excluded sites that were inferred to be heterozygous in at least one of the 162 lines.

To call a putative PTC in the 162 strains, the corresponding reference codon must not be a stop codon. Ancestral states were inferred from the orthologous bases from two outgroup species, that is, *D. sim* and *D. yak*. Syntenic genomic alignments between *D. mel*, *D. sim*, and *D. yak* were downloaded from UCSC (http://hgdownload.soe.ucsc.edu/downloads.html; version dm3 for *D. mel*, droSim1 for *D. sim*, and droYak2 for *D. yak*). We conservatively inferred that a polymorphism was derived in the *D. mel* population if and only if the genome reference strains of *D. mel*, *D. sim*, and *D. yak* all shared the same base. Only such derived variants were used in the subsequent analyses.

One shortcoming of using preassembled pseudochromosomes is that we lost the information on small insertions or deletions (indels). Although indels may affect the calling of PTCs if they occur at neighboring codons, we only observed one case out of the 94 PTC validations, suggesting that such an issue was negligible. For each site, the genotypes of 162 strains were parsed out from their pseudochromosomes according to the SNP’s chromosomal position, and bases other than A, T, G, and C were discarded when calculating the allele frequency spectrum. To maximize sample size while ensuring the accuracy in estimating the DAF, only sites with at least 60 informative bases were used in the subsequent analyses.

To ensure that the patterns, we observed in this study are not specific to the DGRP population, we also applied the same analysis to the *Drosophila* Population Genomics Project 1 database (Langley et al. 2012; Lee and Reinhardt 2012), and the results were in concordance (supplementary table S13 and fig. S13, Supplementary Material online).

### PCR, Sanger Sequencing, and Random Resampling Validation

To experimentally validate the PTCs identified by DGRP, we performed independent Sanger sequencing for 94 randomly chosen PTC sites from each of the three frequency groups: 34 low-, 39 moderate-, and 21 high-frequency PTCs. Primers were designed using Primer3 (version 2.3.4) with the following parameters: 1) PCR products were 300–400 bp long, covering the target sites; 2) the target site was at least 50 bp from either primer; 3) primer length was 20–23 bp with an optimum of 21 bp; 4) melting temperature was 56 to 60 °C with an optimum temperature of 58 °C; 5) GC-content was 40–60% with an optimum level of 50%. All primer pairs were further examined using in-silico PCR to ensure that a single PCR product was produced genome-wide. Then, 116 qualified primer pairs were generated (sequences available upon request). PCR was performed with the following conditions: 94.0 °C for 3min; 35 cycles of the following steps (94 °C for 30 s; 55 °C for 30 s; 72 °C for 1 min); 72 °C for 2 min; 4 °C forever. Finally, the PCR products were sequenced using only the 5′-primers. Raw sequencing results were examined manually on Sequencher (v4.5). Only sites with a single peak were included for analysis. Our results suggest that at least 95% of the 94 PTCs tested are homozygous. Detailed information on our Sanger validation is available in supplementary table S2, Supplementary Material online.

The TPR for low-, moderate-, and high-frequency PTCs were 80%, 87%, and 100%, respectively. With these estimates, we performed resampling by randomly removing putative PTCs from each group using the estimated true positive rate. We generated 100 pseudo-data sets using this procedure and repeated all major analyses. The results are presented in supplementary figures S14–S16, Supplementary Material online, and the major conclusions are robust with respect to false positives.

### Expression and Functional Data Analysis

Raw microarray data for the DGRP lines were retrieved from ArrayExpress (Brazma et al. 2003) with the accession number E-MEXP-1594. The data consist of 160 samples that came from the 40 core DGRP lines, with four replicates—two males and two females—for each line. Based on previous methods (Zhang et al. 2010a), a custom CDF file (version 15.0.0) covering 12,045 genes with unique probes was used, which significantly improved the quality of the array data analysis (Dai et al. 2005). We reanalyzed the microarray data using Bioconductor packages (Gentleman et al. 2004). Briefly, Simpleaffy (v2.32.0) and MAS 5.0 from the affy package (v1.34.0) were used to adjust the background intensity and to normalize and summarize the expression values. From the complete list of PTCs, we chose the subset in which both alleles were present in the core 40 lines. We performed the Wilcoxon rank test to determine the significance of differential expression between the PTC and wild-type alleles. The false discovery rate (FDR) was calculated following that of Storey and Tibshirani (Storey and Tibshirani 2003) with a threshold of 0.05 to call significant differential expression. We applied the same procedure to a randomly chosen, allele frequency-matching set of nonsynonymous and synonymous polymorphisms as a control.

Tissue profiling data were retrieved from FlyAtlas (Chintapalli et al. 2007). We focused on tissue-level expression and therefore excluded adult whole fly, whole larvae feeding, and S2 cells; the redone version of mated adult female spermatheca was also excluded. Raw data were processed using the same pipeline as described above for the DGRP data. For each tissue, the median value of the four replicates was used to represent the expression level of the tissue. R (version 2.15.0) and Microsoft Excel were used to plot and visualize the data. The expression breadth is the number of tissues in which one focal gene is called present by MAS5 across all four replicates. Additionally, we used the index *τ* cutoff of 0.85 to define tissue-specific genes (Yanai et al. 2004). Considering the transcriptional similarity of tissues between larval and adult stages, we only used adult tissues from FlyAtlas. Following a previous study (Yanai et al. 2004), we performed normalization by log_10_ transforming the MAS5 signal values, subtracting the mean of the array of interest and adding the experimental mean across all arrays. To account for the random fluctuation of lowly expressed genes, we only categorized the genes called significantly present by MAS5 across all four replicates into ten equal density quantiles (group 1–10), and the remaining absent genes were assigned to group 0. We then used the quantiled profiles to calculate *τ* based on a formerly described formula (Yanai et al. 2004). Notably, *τ* is not applicable for genes without expression across all tissues because the denominator will become 0 in the original formula.

Processed ModEncode data were retrieved from FlyBase (www.flybase.org) (Drysdale and FlyBase 2008; Graveley et al. 2011). We applied the same analysis pipeline as for FlyAtlas. Finally, the differentially expressed genes (significantly up- or down-regulated) after the loss of ATF3 were obtained directly from the supplementary file, Supplementary Material online of a previous study (Rynes et al. 2012) without further processing.

### Age Dating

Gene age information was obtained from Zhang et al. (Zhang et al. 2010a). CG IDs were used as queries to search the Ensembl website (Flicek et al. 2012) to obtain the corresponding FBgn IDs. Then, all ambiguous IDs were checked individually and manually using the FlyBase website. Eventually, the unambiguous 892 young genes and 11,121 old genes were used for further analysis. The term “new gene” refers to an evolutionarily derived genic locus, which do not need specific age information. The terms young genes and “old genes” refer to genes (in the *D. mel* genome) that originated after and before the split of the subgenera *Sophophora* and *Drosophila*, respectively. The origination mechanism information for DNA- and RNA-level duplicates as well as de novo genes was obtained from a former study (Zhang et al. 2010a). To serve as a control data set of young de novo genes, 5,986 old singleton genes (237 with PTCs) that satisfied the following two criteria were identified: 1) no paralogs exist based on Ensembl homolog annotation and 2) the genes predate the split of the subgenera *Sophophora* and *Drosophila*.

Additionally, 150 genes (11 with high-frequency PTCs, 32 with moderate-frequency PTCs, and 107 with low-frequency PTCs) (supplementary table S6, Supplementary Material online) were manually dated by examining the phylogenetic distribution of orthologs in FlyBase, Ensembl, and UCSC. In FlyBase and Ensembl, we directly used precomputed ortholog annotation. In UCSC, the candidate orthologous sequences in related species were extracted and searched against *D. mel* to ensure the reciprocal relationship. When lacking information in Ensembl and Flybase, Genewise (Birney et al. 2004) was used to align two candidate sequences with a criterion of more than 50% similarity.

The ages of old genes were dated by taking advantage of non-*Drosophila* insect genomes including *Apis mellifera*, *Tribolium castaneum*, *Bombyx mori*, *Anopheles gambiae*, and *Aedes aegypti*. Their phylogeny and branch information with *D. mel* are shown in supplementary figure S17, Supplementary Material online. The gene annotations were downloaded from BeeBase (Elsik et al. 2006), BeetleBase (Wang et al. 2007), SilkDB (Wang et al. 2005), and Ensembl (Hubbard et al. 2007), respectively. The species tree of a previous study (Zdobnov and Bork 2007) was used. Because of the extremely long evolutionary distance spanning this part of the phylogeny, combined with the relatively short generation times for most of the species, the construction of a high-coverage synteny chain between these reference species and *Drosophila* is unlikely (Zdobnov and Bork 2007). In this case, we applied the following procedure to determine orthologous relationships and to date the genes: For *A. gambiae* and *Ae. aegypti*, we used the orthology annotation of Ensembl (Hubbard et al. 2007); for the remaining species, we first identified the reciprocal best hit based on BLAST (Version 2.2.16) results (Altschul et al. 1997). If such a reciprocal best hit exists for gene X, we consider X older than the common ancestor of the two species being compared. This method has potential problems (Koonin 2005). For example, its success is dependent on the quality and coverage of gene annotation; therefore, it is biased against less studied species. In light of the potential inaccuracy in gene dating, we conservatively assigned genes to older branches rather than younger branches. For example, gene X is called as *D. mel* and *A. gambiae*/*Ae. aegypti* lineage-specific only if it is absent in all three outgroup species.

## Supplementary Material

Supplementary tables S1–S13 and figures S1–S17 are available at *Molecular Biology and Evolution* online (http://www.mbe.oxfordjournals.org/).

Supplementary Data
