# Supplementary material for: Expression Profile and Gene Age Jointly Shaped the Genome-Wide Distribution of Premature Termination Codons in a Drosophila melanogaster Population
Source: Mol Biol Evol. 2014 Nov 3;32(1):216–28. doi: 10.1093/molbev/msu299 (PMC4271532; doi:10.1093/molbev/msu299)
Supplement: Supplementary Data [file supp_32_1_216__index.html]

Expression profile and gene age jointly shaped the genome-wide distribution of premature termination codons in a Drosophila melanogaster population — Expression Profile and Gene Age Jointly Shaped the Genome-Wide Distribution of Premature Termination Codons in a Drosophila melanogaster Population — Expression Profile and Gene Age Jointly Shaped the Genome-Wide Distribution of Premature Termination Codons in a Drosophila melanogaster Population — Supplementary Data 

# Expression Profile and Gene Age Jointly Shaped the Genome-Wide Distribution of Premature Termination Codons in a *Drosophila melanogaster* Population

## Supplementary Data

files

**Files in this Data Supplement:**

- Supplementary Data - pdf file
- Supplementary Data - pdf file
- Supplementary Data - xlsx file
