# Supplementary material for: Expression Profile and Gene Age Jointly Shaped the Genome-Wide Distribution of Premature Termination Codons in a Drosophila melanogaster Population
Source: Mol Biol Evol. 2014 Nov 3;32(1):216–28. doi: 10.1093/molbev/msu299 (PMC4271532; doi:10.1093/molbev/msu299)
Supplement: Supplementary Data [file supp_msu299_Supplementary_Figures_S1-S8_v20.pdf]

Supplementary Figures S1 to S8

Supplementary Fig. S1. Derived allele frequency of different types of SNP in *D. melanogaster*.

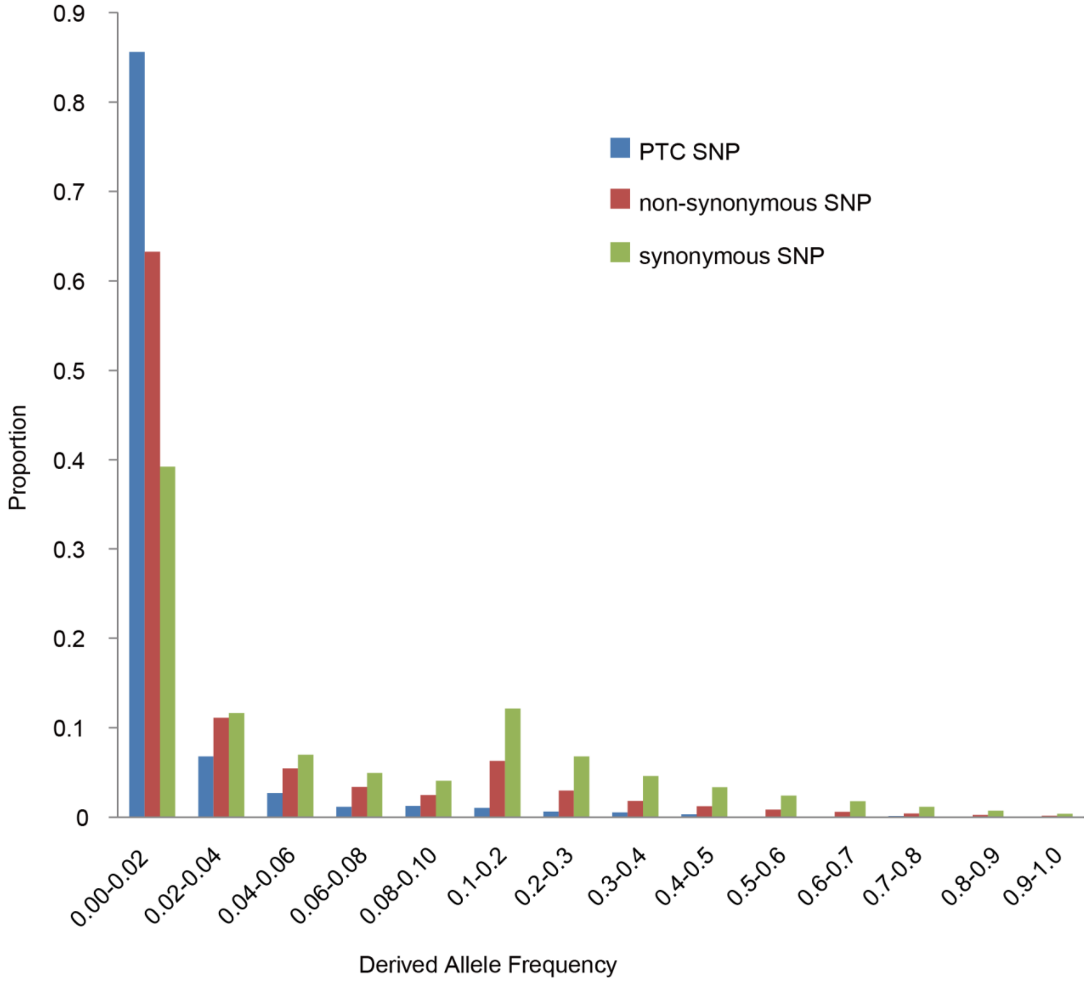

Supplementary Fig. S2. Top expressed tissue for genes with PTCs with different derived allele frequency.

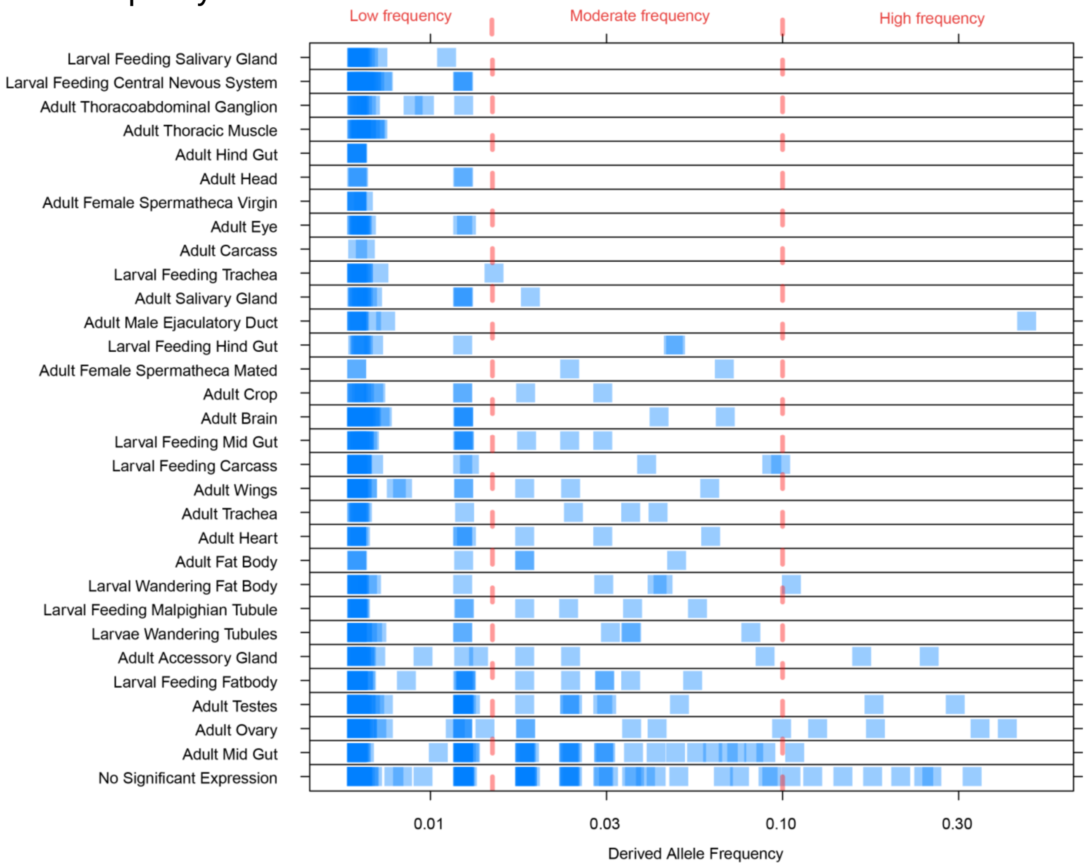

Supplementary Fig. S3. Heatmap of genes encoding moderate- and high-frequency PTCs based on FlyAtlas microarray data.

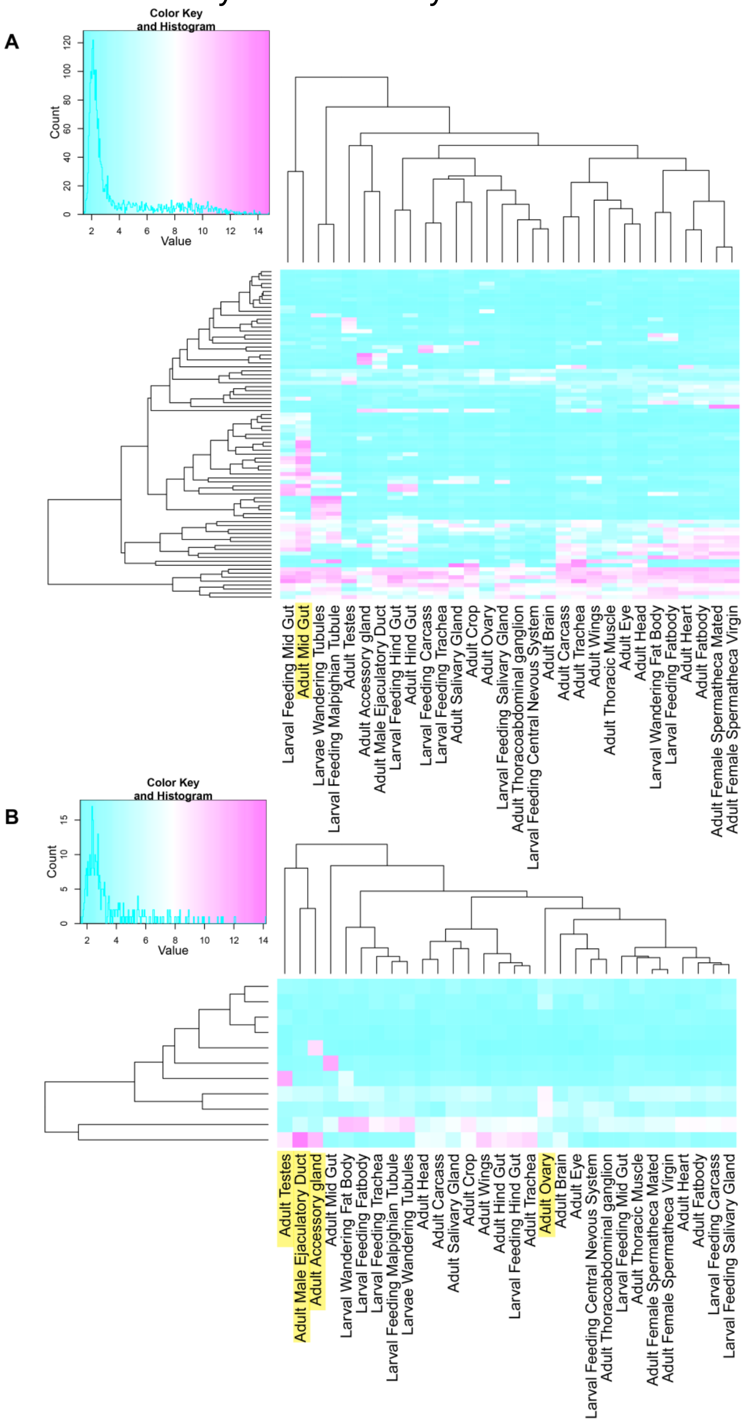

(A)/(B) are expressional intensity of genes harboring moderate/high-frequency PTC respectively. Only genes called present in at least one tissue are shown (Materials and Methods). Adult mid gut and sex related tissues are high-lightend in yellow for (A) and (B), respectively.

Supplementary Fig. S4. Expression profile of PTC-encoding genes after filtering with tissue specificity index (τ) > 0.85.

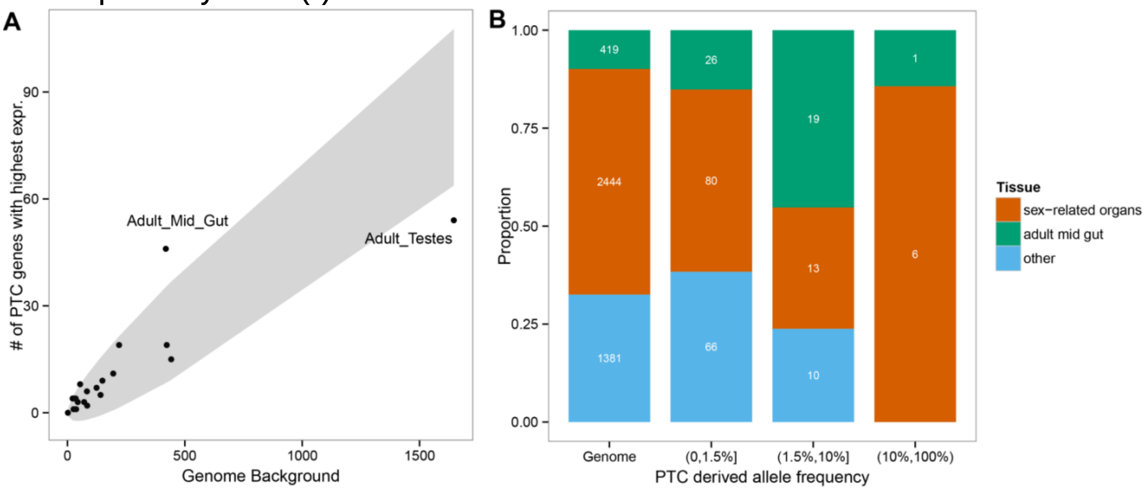

The figure convention follows Fig. 1A & 1B in the main text, except that the PTC-encoding gene set is first filtered by computing the **τ** value (see Materials and Methods) for each gene, and then only including genes with its value > 0.85. This approach excludes genes that have relatively flat expression profiles across tissues and includes tissue-specific genes. See Fig. 1 legend for explanation of the axes and other features of the plots.

Supplementary Fig. S5. Heatmap of genes encoding moderate- and high-frequency PTCs based on modEncode RNA-seq data.

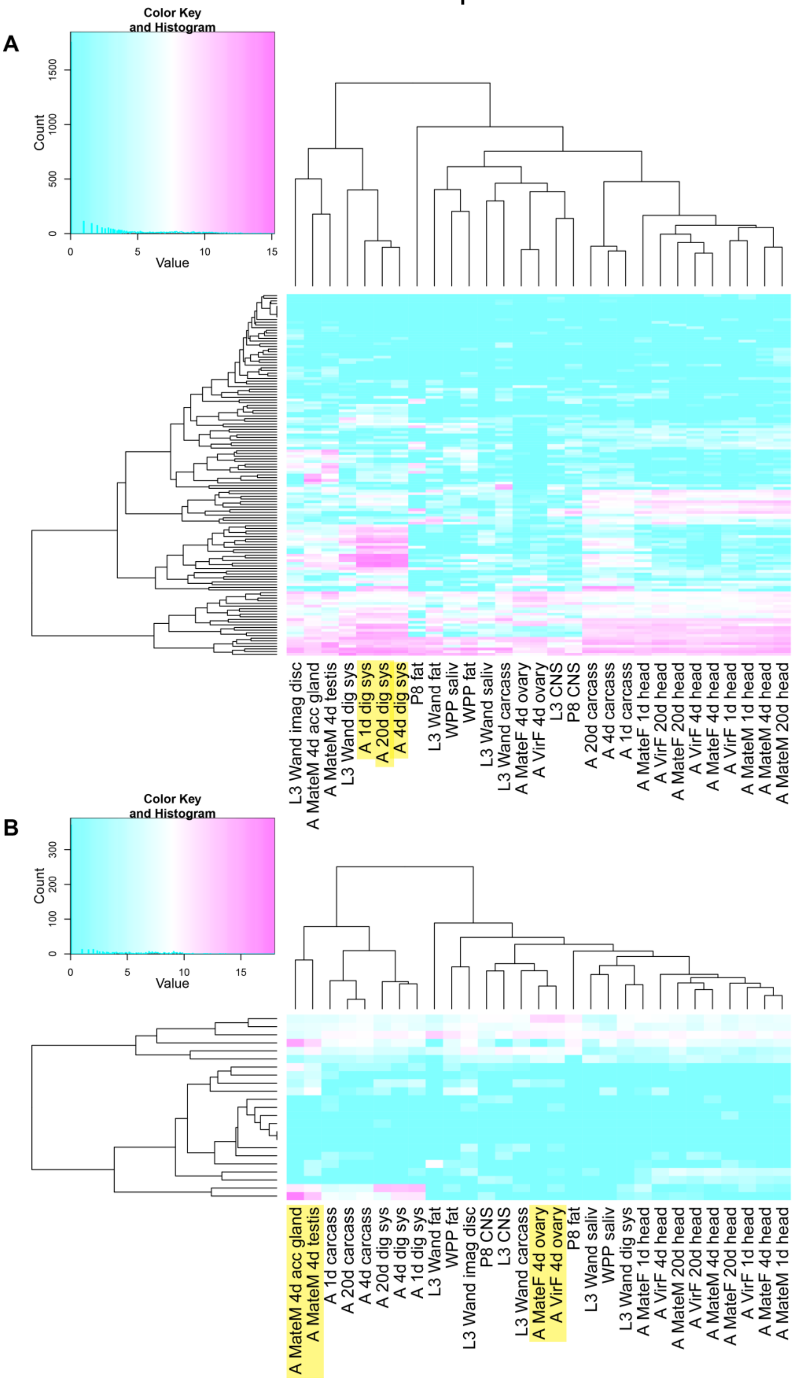

(A)/(B) are $\log_2$(RPKM + 1) values of genes harboring moderate/high-frequency PTC, respectively (Materials and Methods). RPKM refers to “read count per kb per million reads”. Adult digestive system and sex related tissues are high-lightend in yellow for (A) and (B), respectively.

Supplementary Fig. S6. Top expressed tissues of modENCODE RNA-seq data.

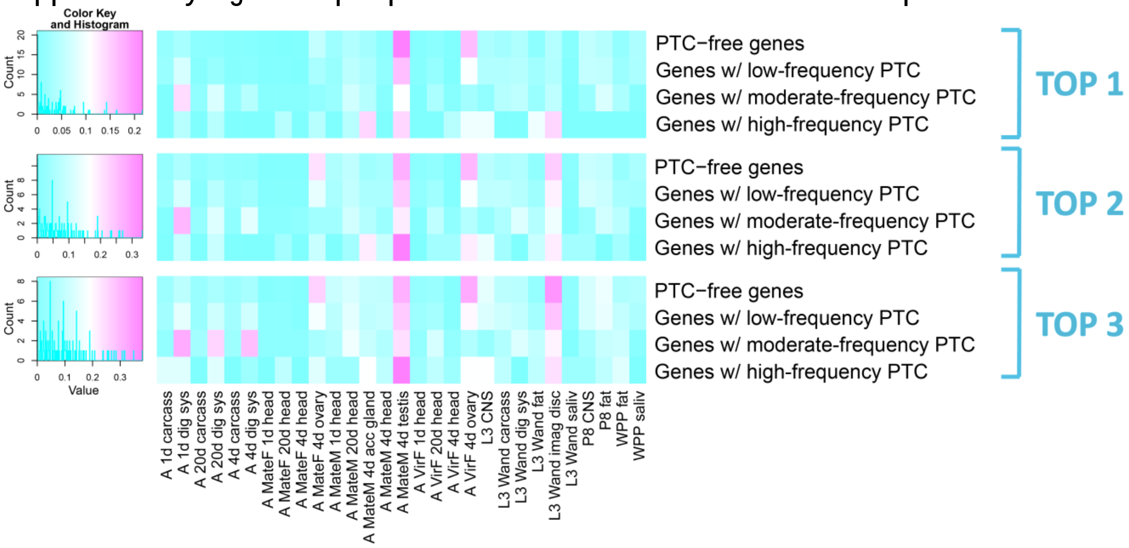

Top expressed tissues are used to generate the distribution of proportions, and three criteria are used which result in a similar pattern. For example, in TOP3, we used the top three highest expressional tissues to calculate the distribution of occurrence, and the same method applies to TOP2 and TOP1 as well. Each cell color coded the proportion of genes with top expression in the focal tissue for one specified category such as PTC-free genes or genes harboring low-frequency PTCs.

Supplementary Fig. S7. Tissue specificity index (τ) distribution for expressed genes in different PTC categories.

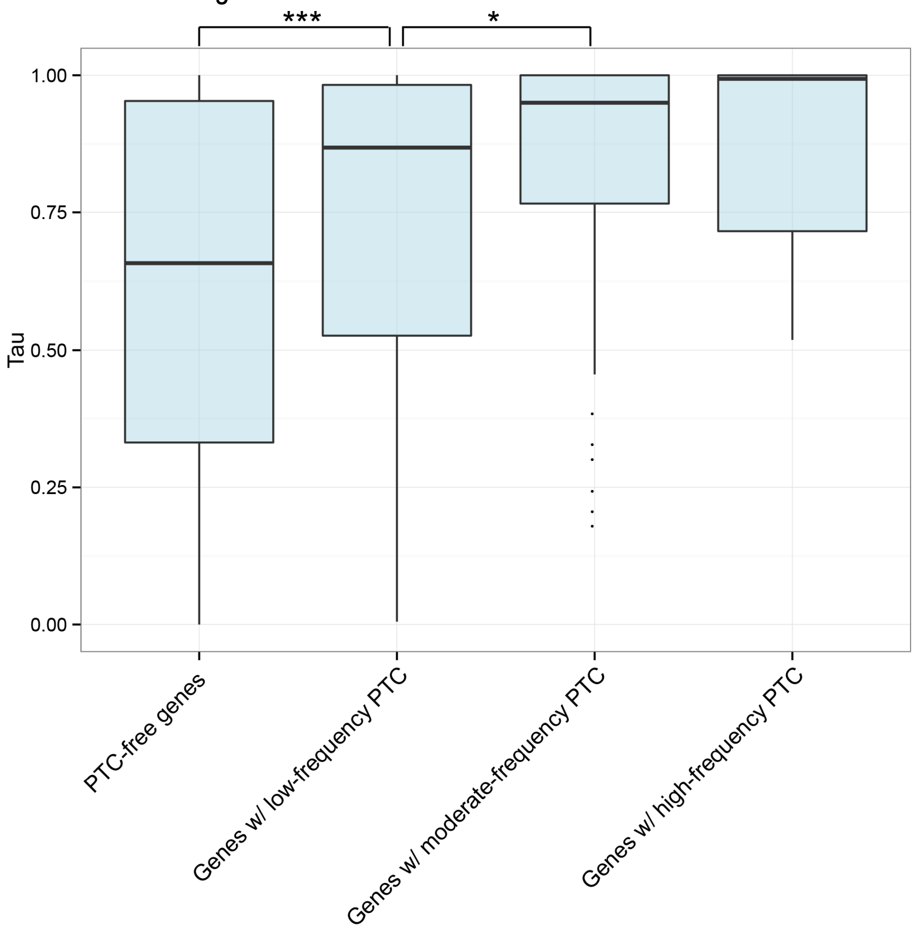

* and *** denote P < 0.05 and P < 0.001 in Wilcoxon rank test.

Supplementary Fig. S8. Percentage of young genes in four categories with manual update of the previous automatic analysis.

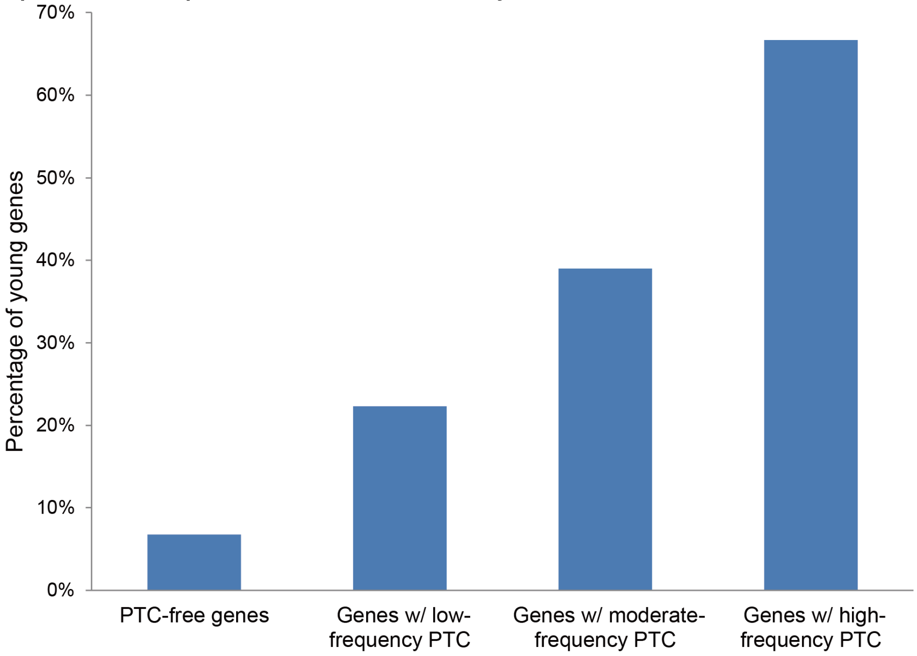

	PTC-free genes	Genes w/ low-frequency PTC	Genes w/ moderate-frequency PTC	Genes w/ high-frequency PTC
**All gene number**	11344	645	136	24
**Old gene number**	10580	501	83	8
**Young gene number**	764	144	53	16
