# Supplementary material for: Expression Profile and Gene Age Jointly Shaped the Genome-Wide Distribution of Premature Termination Codons in a Drosophila melanogaster Population
Source: Mol Biol Evol. 2014 Nov 3;32(1):216–28. doi: 10.1093/molbev/msu299 (PMC4271532; doi:10.1093/molbev/msu299)
Supplement: Supplementary Data [file supp_msu299_Supplementary_Figures_S9-S17_v20.pdf]

Supplementary Figures S9 to S17

Supplementary Fig. S9. Percentage of missing data for young genes in DGRP.

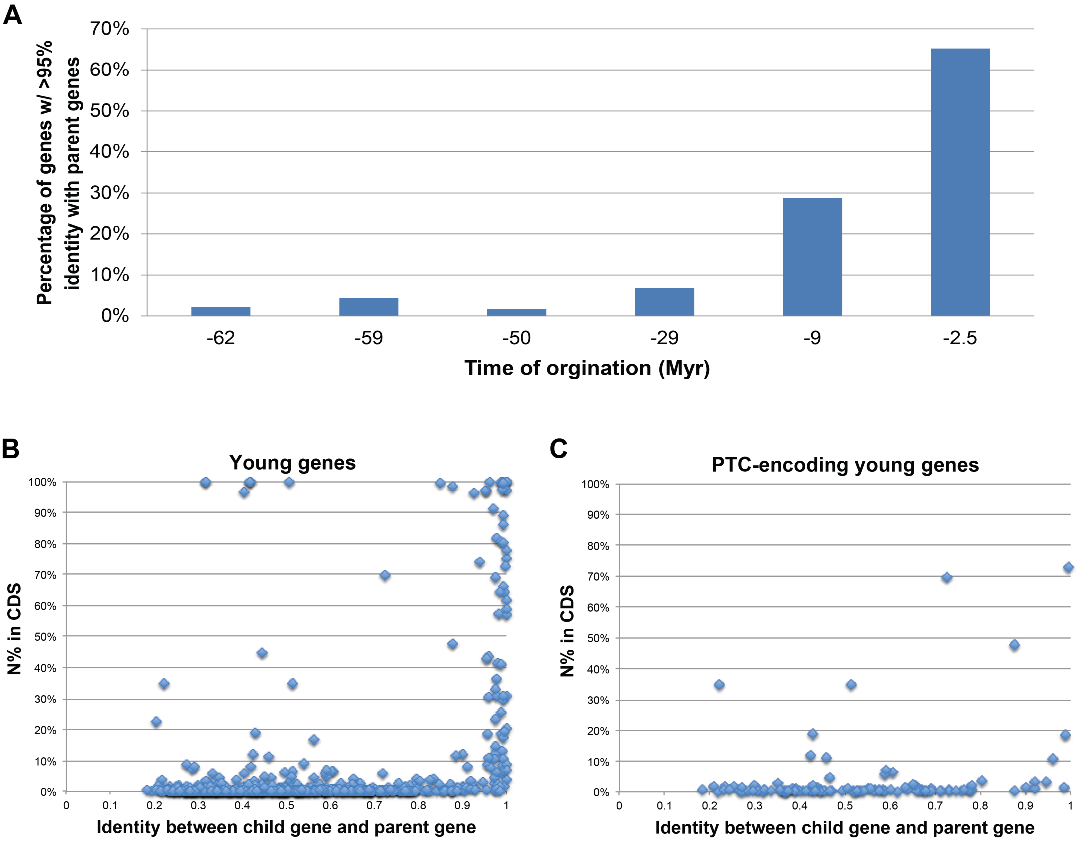

(A) Percentage of young gens with high similarity (> 95% identity) to their parent genes in different age groups. The percentage of "N" in the coding region is calculated for young genes (B) and young genes harboring PTCs (C). In Panel B and C, the mean values across 162 DGRP lines are used for each gene.

Supplementary Fig. S10. Derived allele frequency (DAF) of synonymous substitutions in different age groups.

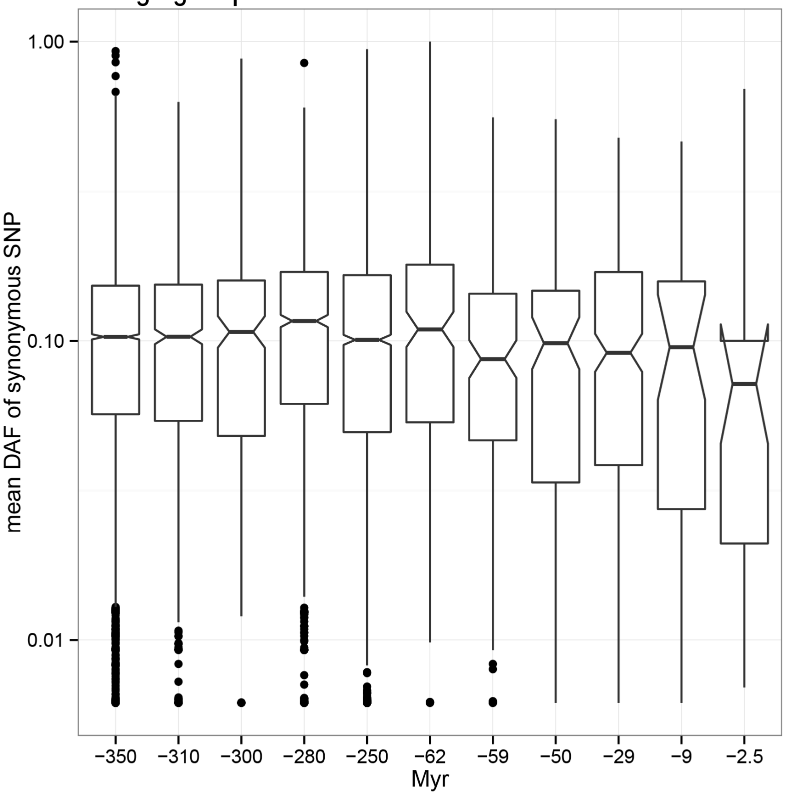

Supplementary Fig. S11. Length of coding sequence for *de novo* genes and retrogenes.

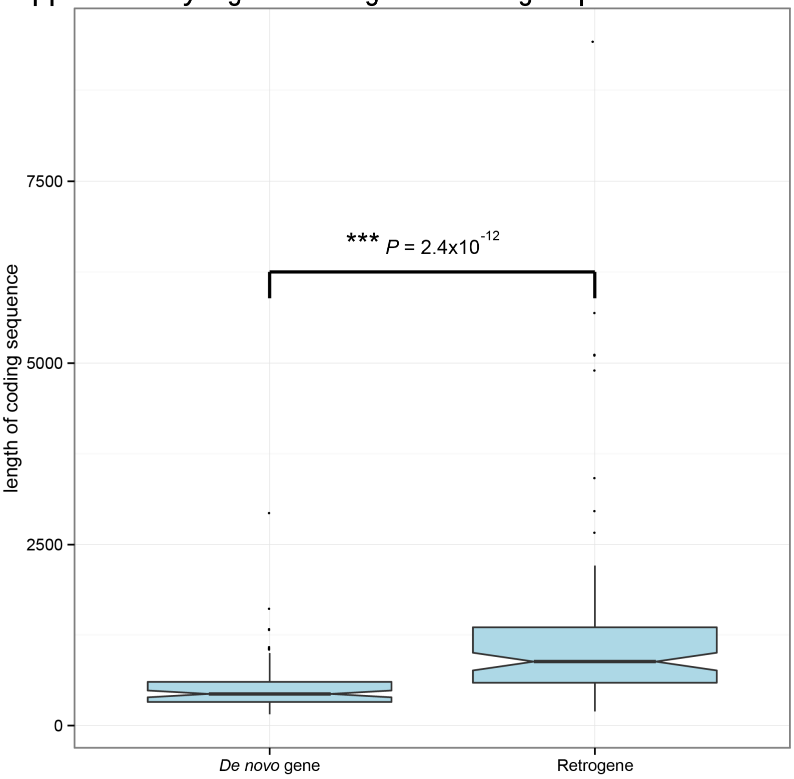

Wilcoxon rank test is used to calculate significance.

Supplementary Fig. S12. Chromosomal distribution of start-lost and splice-lost sites.

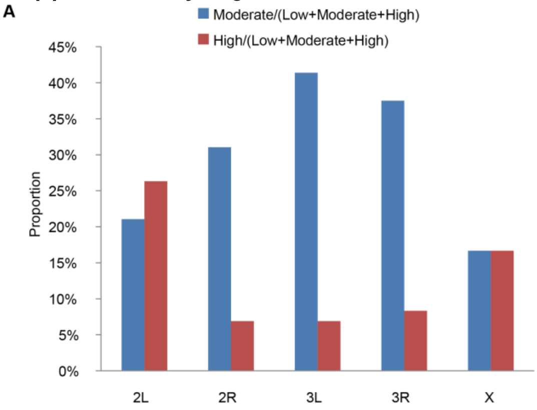

	Start -lost frequency			
	Low+Moderate+High	Low	Moderate	High
2L	19	10	4	5
2R	29	18	9	2
3L	29	15	12	2
3R	24	13	9	2
X	6	4	1	1

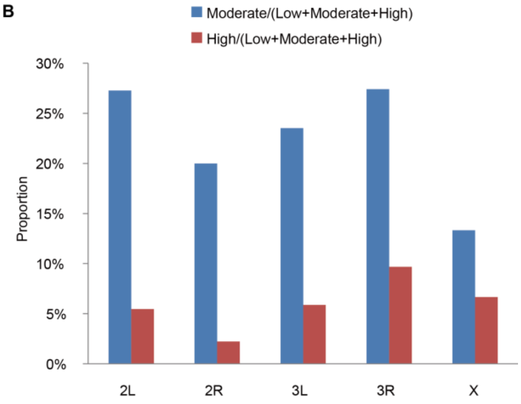

	Splice -lost frequency			
	Low+Moderate+High	Low	Moderate	High
2L	55	37	15	3
2R	45	35	9	1
3L	51	36	12	3
3R	62	39	17	6
X	15	12	2	1

Numbers of start-lost sites (A) and splice-lost sites (B) on different chromosomes.

Supplementary Fig. S13. Percentage of genes harboring PTC mutations within each age group by using previous gene loss data (Lee and Reinhardt 2012).

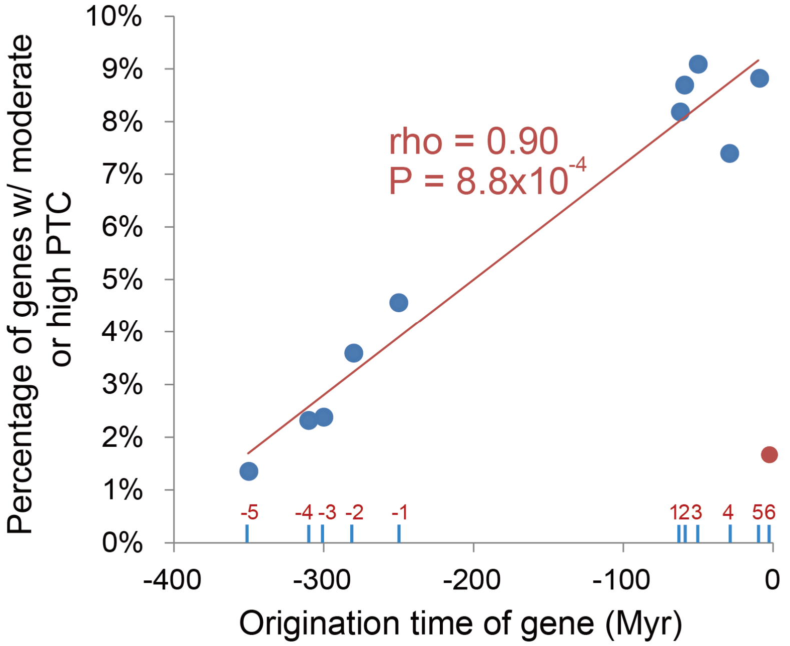

Supplementary Fig. S14. A sample plot from 100 random resampling results for expression profile of PTC-encoding genes (related to Fig. 1A & 1B).

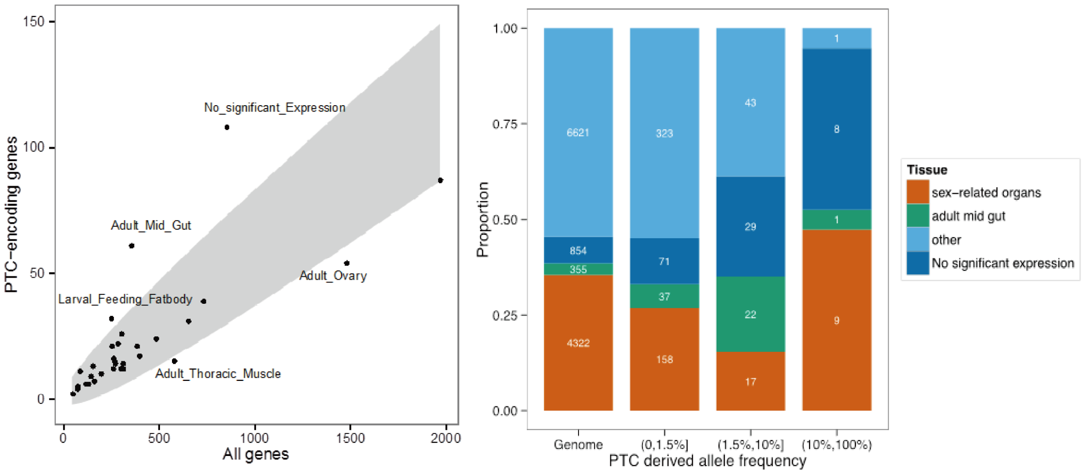

Tissue	# of times significant in 100 pseudo samples	Direction (excess of PTC vs. deficit of PTC)
Adult_Mid_Gut	100	Excess
No_significant_Expression	100	Excess
Larval_Feeding_Fatbody	90	Excess
Adult_Ovary	87	Deficit
Adult_Thoracic_Muscle	53	Deficit
Adult_Testes	24	Deficit
Adult_Trachea	8	Excess

The above figures are a sample plot from one of the 100 pseudo-datasets randomly resampled based on the false positive rates of low-, moderate- and high- frequency PTCs, respectively. Among all 100 pseudo datasets, we found highly similar patterns for results presented in Fig. 1A, as listed in the above table (# of times significant in the 100 resampling tests). For results presented in Fig. 1B, we tested the differences between the three PTC frequency groups with the genome background in the 100 pseudo-datasets. After controlling for multiple testing, all three tests were significant at 0.01 level.

Supplementary Fig. S15. A sample plot from one of the 100 random resampling results for the expression breadth differences among the three frequency groups (related to Fig. 2A)

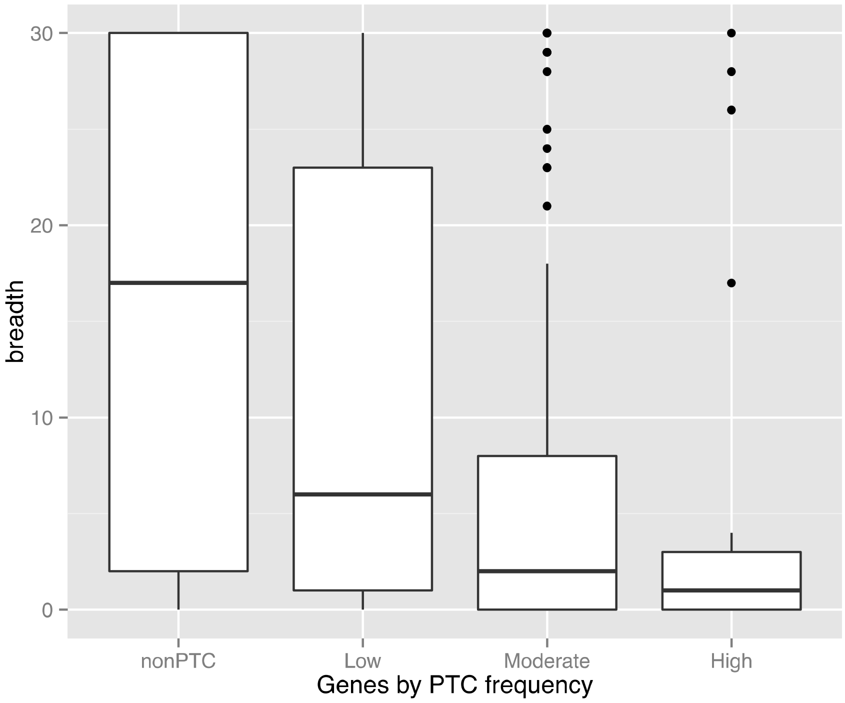

The above figure is a sample plot from one of the 100 pseudo-datasets randomly resampled based on the false positive rates of low-, moderate- and high- frequency PTCs, respectively. In all 100 pseudo-datasets, we tested and found the differences between the non-PTC *vs.* low-frequency PTC and low-frequency *vs.* moderate-frequency to be significant at 0.01 level after correcting for multiple testing.

Supplementary Fig. S16. 100 random sampling results for percentage of genes with PTC sites in each age group (related to Fig. 3B & 3C).

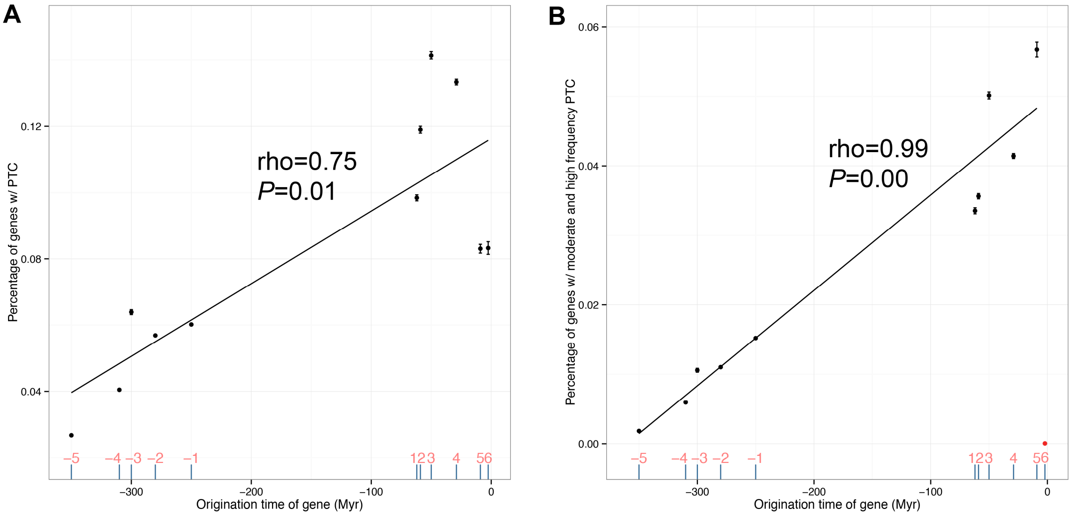

(A) Percentage of PTC-encoding genes in each age group; (B) percentage of genes harboring moderate or high-frequency PTCs in each age group. 100-time random samplings are conducted by controlling the error rate of PTCs (20%, 13% and 0% for low, medium and high-frequency, respectively). Linear regression is used in Panel A (all points) and Panel B (the youngest group is excluded and marked in red). The rho and *P* values are from the Spearman test of the mean percentage values. The error bars show the standard errors of the mean percentage values.

Supplementary Fig. S17. Dating of old genes with five non-*Drosophila* insects as outgroups

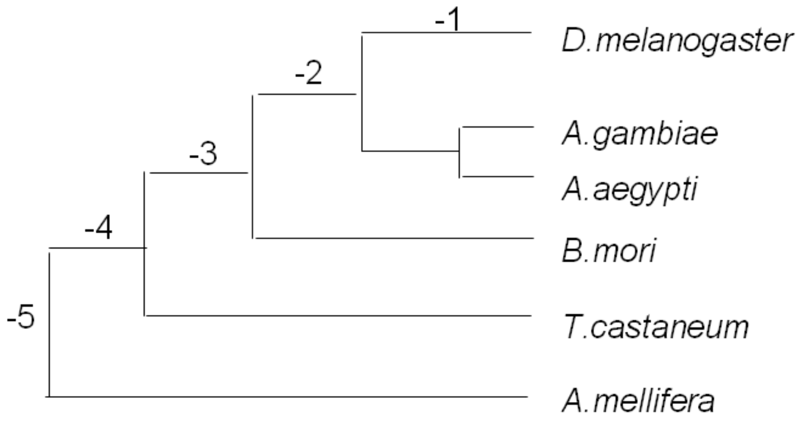

We partitioned the old genes (Zhang et al. 2010) into five groups, branch -1 to -5. For example, branch -1 indicates genes occurring just after the divergence of *Drosophila* group and *A.gambiae*/*A.aegypti* and before the split of *Drosophila* genus.

Reference for Supplementary Figures

Lee YC, Reinhardt JA. 2012. Widespread polymorphism in the positions of stop codons in Drosophila melanogaster. *Genome Biol Evol* 4(4): 533-549.

Zhang YE, Vibranovski MD, Krinsky BH, Long M. 2010. Age-dependent chromosomal distribution of male-biased genes in Drosophila. *Genome Res* 20(11): 1526-1533.
